# Extraocular Muscle Characteristics Related to Myasthenia Gravis Susceptibility

**DOI:** 10.1371/journal.pone.0055611

**Published:** 2013-02-08

**Authors:** Rui Liu, Hanpeng Xu, Guiping Wang, Jie Li, Lin Gou, Lihua Zhang, Jianting Miao, Zhuyi Li

**Affiliations:** 1 Department of Geratology, Tangdu Hospital, Fourth Military Medical University, Xi'an, Shaanxi Province, P. R. China; 2 LONI, Department of Neurology, UCLA, Los Angeles, California, United States of America; 3 Department of Neurosurgery, 208th Hospital of PLA, Changchun, Jilin Province, P. R. China; 4 Department of Endocrinology, 451 Hospital of PLA,Xi'an, Shaanxi Province, P. R. China; 5 Department of Neurology, Tangdu Hospital, Fourth Military Medical University, Xi'an, Shaanxi Province, P. R. China; Charité University Medicine Berlin, Germany

## Abstract

**Background:**

The pathogenesis of extraocular muscle (EOM) weakness in myasthenia gravis might involve a mechanism specific to the EOM. The aim of this study was to investigate characteristics of the EOM related to its susceptibility to myasthenia gravis.

**Methods:**

Female F344 rats and female Sprague-Dawley rats were assigned to experimental and control groups. The experimental group received injection with Ringer solution containing monoclonal antibody against the acetylcholine receptor (AChR), mAb35 (0.25 mg/kg), to induce experimental autoimmune myasthenia gravis, and the control group received injection with Ringer solution alone. Three muscles were analyzed: EOM, diaphragm, and tibialis anterior. Tissues were examined by light microscopy, fluorescence histochemistry, and transmission electron microscopy. Western blot analysis was used to assess marker expression and ELISA analysis was used to quantify creatine kinase levels. Microarray assay was conducted to detect differentially expressed genes.

**Results:**

In the experimental group, the EOM showed a simpler neuromuscular junction (NMJ) structure compared to the other muscles; the NMJ had fewer synaptic folds, showed a lesser amount of AChR, and the endplate was wider compared to the other muscles. Results of microarray assay showed differential expression of 54 genes in the EOM between the experimental and control groups.

**Conclusion:**

Various EOM characteristics appear to be related to the increased susceptibility of the EOM and the mechanism of EOM weakness in myasthenia gravis.

## Introduction

Myasthenia gravis (MG) is characterized by muscle weakness and fatigability, which is attributed to the presence of autoantibodies against the acetylcholine receptor (AChR), leading to dysfunction of acetylcholine signal transduction [1]. However, in patients with ocular MG, the autoantibody titer is relatively low [2]. The extraocular muscles (EOM) are known to be susceptible in other autoimmune diseases such as Grave disease [3], suggesting the presence of a unique mechanism that dictates pathogenesis of EOM weakness in MG and other autoimmune diseases, which is not present in other skeletal muscles. There are relatively few studies investigating the mechanism of EOM weakness, a common manifestation in patients with MG.

Previous studies have revealed that EOM expresses the embryonic form of the AChR, which is different from the AChR found in skeletal muscle [4]. However, it is not clear if embryonic AChR is a target of immune system attack resulting in EOM weakness in patients with MG. In fact, patients with MG who experience EOM weakness do not have circulating antibodies against embryonic AChR or T-cell immunity specific to embryonic AChR [5]. Additionally, active or passive immunity induced by the embryonic AChR or its antibody does not induce symptoms of MG. These findings suggest the presence of specific characteristics that predispose the EOM to susceptibility to damage, making it a target in MG patients.

The EOM has special functions. Contraction is rapid, and there is a remarkable tolerability to fatigue. Under normal conditions, it is easy to maintain stable contraction of the EOM in the presence of high-frequency nerve impulses. Previous studies showed differential expression of a number of genes including immune response genes and complement genes in EOM, suggesting that EOM could be particularly susceptible to complement-mediated injury triggered in MG [6]. In the present study, we aimed to investigate factors associated with increased susceptibility of EOM in MG. In particular, based on factors that decrease the safety of the neuromuscular junction (NMJ) [7], we investigated the mechanism of EOM weakness in MG.

## Materials and Methods

### Animals and Preparation of AChR Monoclonal Antibody (mAb35)

All animal procedures were approved by the Ethics Committee of Tangdu Hospital, Fourth Military Medical University, and the protocols followed the ethical guidelines of this committee. Nude mice purchased from Animal research center, Fourth Military Medical (Shaanxi, China) were housed one per cage in our animal facility, with a 12∶12 h light-dark cycle and ad libitum access to food and water.

The TIB175 hybridoma cell line (American Type Culture Collection, Manassas, VA) was maintained in Dulbecco modified Eagle medium (Life Sciences Corp., Grand Island, NY) containing 10% fetal bovine serum. Nude mice were intraperitoneally inoculated with cells (1×10^6^ cells/mouse) to generate the mAChR antibody *(mAb35)*. One week after inoculation, ascites were collected, and IgG was extracted by the ammonium sulfate salting-out method. After dialysis, the Lowry method was used to determine protein concentration. IgG was subjected to 15% SDS-PAGE and the purity was 97%.

Female F344 rats aged 10 to 11 weeks (∼160 g) were purchased from Shanghai Slaccas Laboratory Animal Co. (Shanghai, China) and used for microarrays according to previous reports [8]. Female Sprague-Dawley (SD) rats aged 10 to 11 weeks (∼200 g) were purchased from the Animal Center of the Fourth Military Medical University. Rats were assigned to the experimental group or the control group. The experimental group received an intraperitoneal injection of Ringer solution (1 ml) containing mAb35 (0.25 mg/kg) to induce experimental autoimmune myasthenia gravis as previously described [9], [10], while the control group received an intraperitoneal injection of Ringer solution alone. The animal facility had a 12∶12 h light-dark cycle. Three rats were housed per cage and had ad libitum access to food and water for 48 h before experiments.

### Assessment of EOM Symptoms and EOM Wet Weight

Eyeball movement and concomitant symptoms were observed. Rats were sacrificed two days after immunization and the EOM was collected and weighed (wet weight).

### Determination of Creatine Kinase Activity in Serum

Blood was obtained from the orbital sinus of anesthetized mice and allowed to clot. After centrifugation, the serum was collected and stored at −70°C for creatine kinase determinations. Creatine kinase (CK) activity of serum was assessed using the appropriate ELISA test kit (Uscn Life Science, Wuhan,China No. E90109Ra) according to the manufacturer’s instructions. Final results were read at a wavelength of 450 nm.

### Tissue Staining for Light Microscopy

Sprague-Dawley rats were anesthetized with intraperitoneally administered 1% sodium pentobarbital (50 mg/kg) and were then perfused transcardially with 150 ml normal saline. Muscles (EOM, diaphragm, and tibialis anterior) were collected. For staining of succinate dehydrogenase (SDH), muscles were stored in liquid nitrogen with no antifreeze. For acetylcholinesterase (AChE) staining, rats were perfused transcardially with 150 ml normal saline and then with 500 ml 4% paraformaldehyde. Muscles were collected and fixed in 4% paraformaldehyde again for 2 h, and then placed in 25% sucrose for 24 h until they sank, in order to prevent intracellular ice crystal formation. The following were used for section generation (25-µm thickness): paraffin sections, RM 2135 (Leica; Nussloch, Germany); cryostat sections, CM1900 (Leica); vibrating blade microtome sections, VT1000S (Leica); ultramicrotome sections, LKB Nova (LKB, Stockholm, Sweden). Sections were placed onto uncoated slides.

For SDH staining, sections were incubated in SDH buffer (50 ml 0.2 M sodium succinate, 50 ml 0.2 M phosphate buffer, 100 mg nitroblue tetrazolium chloride) at room temperature for 1 h and fixed in 4% paraformaldehyde for 30 min. For AChE staining, sections were incubated in AChE buffer (0.5 ml of 0.1 mM sodium acetate, 1 ml of 30 mM copper sulphate; 5 mg/ml acetylthiocholine iodide, 1 ml of 5 mM potassium ferricyanide, 6.5 ml of 0.1 M PBS) at room temperature for 1 h. For double-staining, SDH–stained sections were also stained for AChE. Sections were observed under an upright, bright-field microscope (BX-60; Olympus, Tokyo, Japan). A total of 10 fields/animal/muscle were randomly selected for analysis. F344 as well as SD rats showed similar pathological changes (data not shown).

### AChR Fluorescence Histochemistry

Free-floating muscle sections were treated with biotin-conjugated α-bungarotoxin (α-BuTx-Biotin; 1∶500; Molecular Probes, Eugene, OR) at room temperature overnight and then with 1∶500 avidin-conjugated Texas Red (Sigma-Aldrich, St. Louis, MO) or avidin-conjugated fluorescein isothiocyanate (FITC-avidin) (Sigma-Aldrich) at room temperature for 3 h. Sections were mounted onto slides with 50% glycerol in 0.1 M phosphate-buffered saline (PBS) and observed under a microscope (BX-51, Olympus; 100 W halogen lamp, 400–700 nm). Representative images were acquired with a digital camera (DP-70, Olympus). The fluorescence exposure time for each field was 20 seconds. We acquired all images first and measured fluorescence intensity later to avoid/decrease any bleaching effect.

### Pre-embedding Immunocytochemistry

Samples were collected and fixed in buffer (0.25% glutaraldehyde, 4% polyoxymethylene, 15% saturated picric acid in pH 7.4, 0.1 mol/l PBS) for 2 h. Samples were then cut into 50-µm sections and treated with α-BuTx-Biotin and avidin-biotin complex (ABC, Vector Laboratories, Burlingame, CA) followed by visualization with diaminobenzidine (DAB) (1∶200, final concentration 200 µg/ml). Sections were immersed in 25% sucrose for 2 h and washed in PBS. After postfixation in 0.5% osmium tetroxide in water at room temperature for 1 hour, sections were dehydrated in an ethanol series and then incubated in propylene oxide and subjected to flat-embedding in epoxide resin (Diglycidyl Ether of Bisphenol-A or DGEBA) with heat. Appropriate regions were selected and glued to a blank block for ultrathin sectioning (500 nm sections, diamond knife, coated copper grid) followed by lead-uranium treatment for contrast. Sections were examined by electron microscopy (JEM100SX, Jeol Ltd., Tokyo, Japan; 60–80 kV accelerating voltage, image recording on film).

### Determination of the Ratio of Postsynaptic Membrane to Presynaptic Membrane

Sprague-Dawley rats were anesthetized and perfused transcardially with 150 ml normal saline, followed by fixation and processing for electron microscopy as described above, with the exception of α-BuTx-Biotin, ABC, and DAB staining. The area and length of the presynaptic membrane and postsynaptic membrane were measured as described previously [11].

### Western Blot Analysis

Agrin and SV2 expressions were detected by Western blot according to the method described previously [12]. In brief, tissue homogenates were prepared by homogenization in a modified RIPA buffer (0.1 M phosphate-buffered saline (PBS)/1% NP-40/0.5%, Tween-20/0.1% sodium dodecyl sulfate (SDS), and complete cocktail inhibitor (Roche, Mannheim, Germany), and extracted on ice for 30 min. Protein concentrations were determined by BCA assay (Pierce, Rockford, IL). A 40 µg/well protein was fractionated on 7.5% SDS-PAGE gels, and transferred to Immobilon P (Millipore, Bedford, MA), then blocked with blocking buffer (Tris-buffered saline, pH 7.6 and 0.5% skimmed milk) and incubated with monoclonal anti-agrin antibody (1∶500; Chemicon, Temecula, CA ) and monoclonal anti-synaptic vesicle protein 2 (anti-SV2) antibody (1∶400; K. Buckley, Harvard Medical School, Boston, MA) at 4°C overnight. The membranes were then incubated with HRP-conjugated anti-mouse antibodies at room temperature for 1 h. The bound antibodies were visualized using LumiGLo reagent (Pierce, Rockford, IL) and the expression levels of SV2 and Agrin relative to β-actin were determined.

### Detection of Differentially Expressed Genes by Microarray Assay

Total RNA was extracted from F344 muscles with TRIzol (Life Technologies Corp.), according to the manufacturer’s instructions. mRNA was purified with an Oligotex mRNA Midi Kit (Qiagen, Chatsworth, CA) and cDNA was prepared by Reverse-transcription Kit according to the manufacturer’s instructions (Shanghai Union Gene Co., Ltd., Shanghai, China). cDNA samples from the control and experimental groups were labeled with Cy3-dUTP and Cy5-dUTP and hybridized to a whole rat Genome Oligo Microarray (BioStarR-40S) from Shanghai Union Gene Co., Ltd. (Shanghai, China) according to the manufacturer’s instructions. A ScanArray 3000 (General Scanning, Moorpark, CA) was used to scan the microarray, and Imagene 3.0 software was used to detect the intensity of Cy3 and Cy5 fluorescence signals. The ratio of Cy5 to Cy3 signals (Cy5/Cy3) was calculated and gene expression was normalized to an internal reference. A gene with Cy5/Cy3 of >2 or <0.5 was defined as a differentially expressed gene. To minimize individual variation, common differentially expressed genes were selected from two microarray assays for analysis.

### Statistical Analyses

Statistical analyses were performed with SPSS 15.0 statistics software (SPSS Inc, Chicago, IL). Comparability of the proportion of muscle fibers between the control and experimental groups was tested by chi-square test, and data are presented as number (*n*) and percent (%). Normally distributed continuous variables were compared by one-way analysis of variance (ANOVA) if there were more than two groups. When a significant difference between groups was apparent, multiple comparisons of means were performed with the Bonferroni procedure, along with type-I error adjustment. In addition, independent two-sample *t* tests were performed to determine the difference between the control and experimental groups. Data are presented as mean ± standard deviation (SD). All statistical assessments were two-sided and evaluated at the 0.05 level of significant difference.

## Results

### Assessment of Ocular Symptoms and EOM Wet Weight

The specificity of mAb35 was demonstrated in TE671 cells which express skeletal muscle nAchR and in rat brain tissue (data not shown). Circulating AchR antibodies were also detected in rats injected with mAb35, and undetectable in unimmunized animals (data not shown). Anti-cholinesterase inhibitor (neostigmine) relieved MG symptoms while a non-depolarizing muscle relaxant (vecuronium bromide) exacerbated MG symptoms (data not shown). Additionally, comparison of amplitude after stimulation with electrical pulses showed a significant reduction in amplitude in the experimental group compared to the control group (data not ahown).

**Figure 1:**
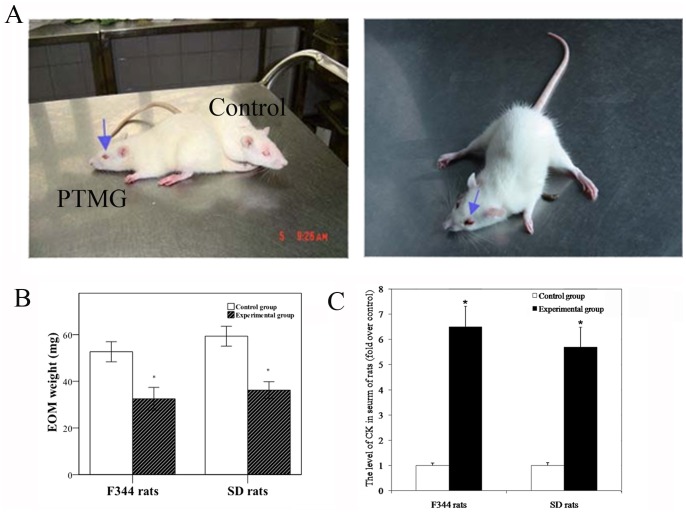
A. Ocular symptoms in PTMG rats. B. Wet weight of the extraocular muscles (EOM) in the control group and experiment group. C. Level of creatine kinase (CK) in the serum determined by ELISA. *Significant (*P*<0.05) difference between the control and experimental groups determined by independent two-sample *t* test.

All PTMG rats exhibited decreased activity and increased lassitude and lethargy compared to control rats. Eye movement was decreased, and the palpebral fissure was small. Rats with severe MG exhibited enophthalmos, congestion, and even bloody discharge. Figure 1A depicted the ocular symptoms of PTMG rats. The experimental groups of both SD and F344 rats had significantly lower EOM wet weights compared to the control groups (Fig. 1B). Serum creatine kinase (CK) levels is a well defined marker for muscular damage which is released into the bloodstream following muscle cell damage or degeneration [13]. http://www.sciencedirect.com/science/article/pii/S0304394003011121 - BIB5The ELISA results showed that the CK levels of serum samples were roughly 6.5-fold higher than the control values (Fig. 1C). The results provide evidences for muscular damage in MG.

### Assessment of the Proportion of Muscle Fibers in Different Muscles

SDH staining was used to compare the proportion of three muscle fiber types in muscles in the control and experimental groups (Table 1). There were significant (*P*<0.05) differences in the proportion of the three types of muscle fibers in EOM and diaphragm muscle between the control and experimental groups. There was also a significant (*P*<0.05) difference between the groups in the proportion of type I and type IIA fibers in tibialis anterior muscle but no significant difference in the proportion of type IIB muscle fibers (*P* = 0.079).

**Table 1:** Comparison of the proportion of three types of muscle fibers in muscles in the two groups.

Muscle Fiber	Control group	Experimental group	*P* value
Extraocular, *n* (%)			
Type I	591 (22.8)	394 (15.1)	<0.001*
Type IIA	781 (30.1)	1093 (41.9)	<0.001*
Type IIB	1221 (47.1)	1123 (43.0)	0.003*
Diaphragm, *n* (%)			
Type I	257 (12.9)	126 (5.5)	<0.001*
Type IIA	798 (39.9)	1023 (44.7)	0.002*
Type IIB	945 (47.3)	962 (42.0)	0.001*
Tibialis anterior, *n* (%)			
Type I	243 (12.8)	174 (9.1)	<0.001*
Type IIA	770 (40.6)	899 (47.1)	<0.001*
Type IIB	885 (46.6)	836 (43.8)	0.079

* Significant (*P*<0.05) difference between the two groups by chi-square *t* test.

### Assessment of Endplate Distribution in Different Muscles

**Figure 2:**
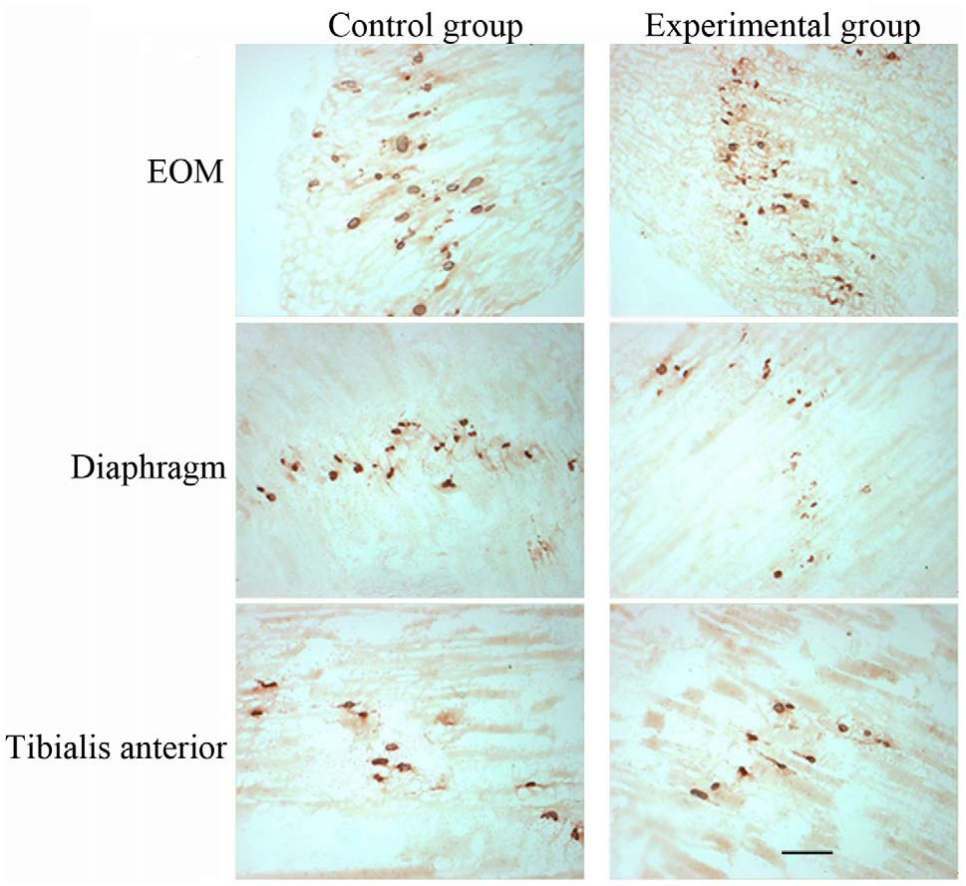
Acetylcholinesterase-positive endplates of three muscles in the two groups assessed by acetylcholinesterase (AChE) staining. Scale bar = 50 µm.

Endplate distribution was assessed by AChE staining. At low magnification, the NMJ was found to be arranged in the middle segment of muscle fibers of different muscles, forming the endplate. The endplate was compared in different muscles. Results showed that the endplate of the EOM was wider than that of the diaphragm or tibialis anterior in both control and PTMG groups (Fig. 2). In addition, some small NMJs were also distributed in the EOM. In the experimental group, the NMJ of the endplate of different muscles was slightly smaller than those in the control group. We also found that the endplate of the EOM was significantly narrowed, but this change was not obvious in the diaphragm and tibialis anterior muscles (Fig. 2).

### Comparison of NMJs from Different Muscles

**Figure 3:**
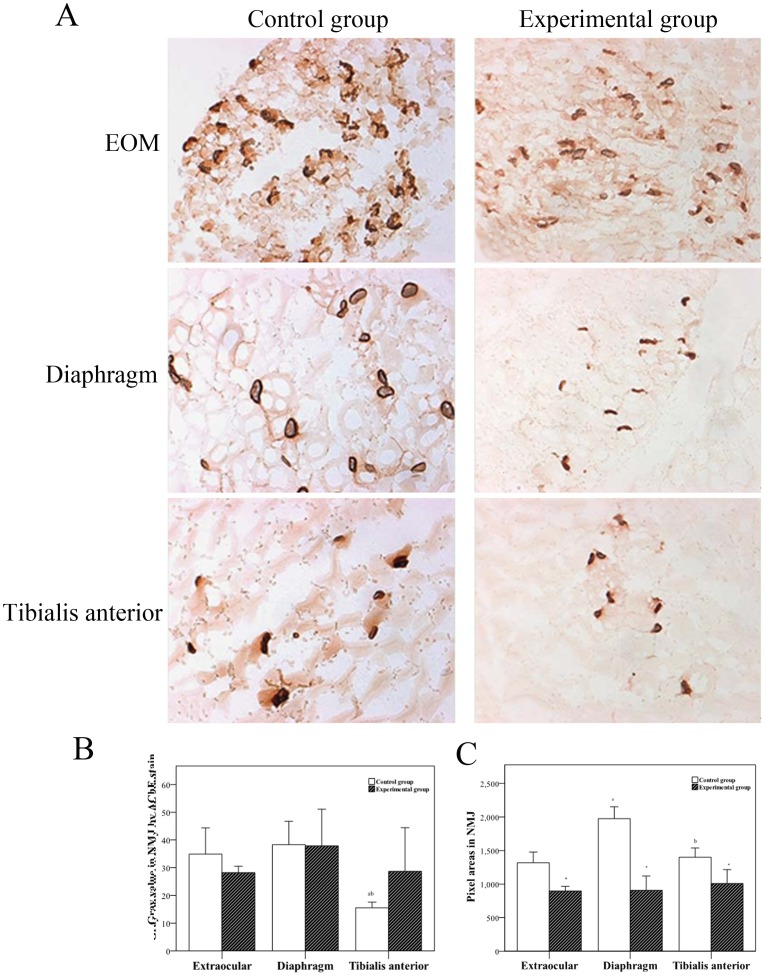
Comparision of NMJs from different muscles. A. Acetylcholinesterase (AChE) staining of three muscles in the two groups. B. Determination of gray value in NMJ by an automatic analyzer (DM2500,Leica,Germany). C. Determination of pixel areas in NMJ by an automatic analyzer. *Significant (*P*<0.05) difference between control and experimental groups determined by independent two-sample *t* test. ^a^Statistically significant difference between indicated group and EOM group. ^b^Statistically significant difference between indicated group and diaphragm group. Scale bar = 20 µm.

AChE staining showed that the NMJ was brown, flat, or oblate and distributed along the border of the muscle fibers (Fig. 3). The size of the AChE–positive region reflected the size of the NMJ, and the density of this region reflected the AChE content. The NMJ area was smaller in the experimental groups when compared to the control groups, and this decrease was more obvious in the EOM than in diaphragm and tibialis anterior muscles. However, the AChE content remained largely unchanged (Fig. 3A). There was also a significant decrease in the NMJ area in different muscles in the experimental group compared to the control group. In the control group, the NMJ area of diaphragm muscle was slightly larger than that of EOM and tibialis anterior muscle (Fig. 3B). The AChE content in the NMJ was comparable between the experimental and control groups (Fig. 3C).

### Comparison of AChRs in the NMJ of Different Muscles by Fluorescence Histochemistry

**Figure 4:**
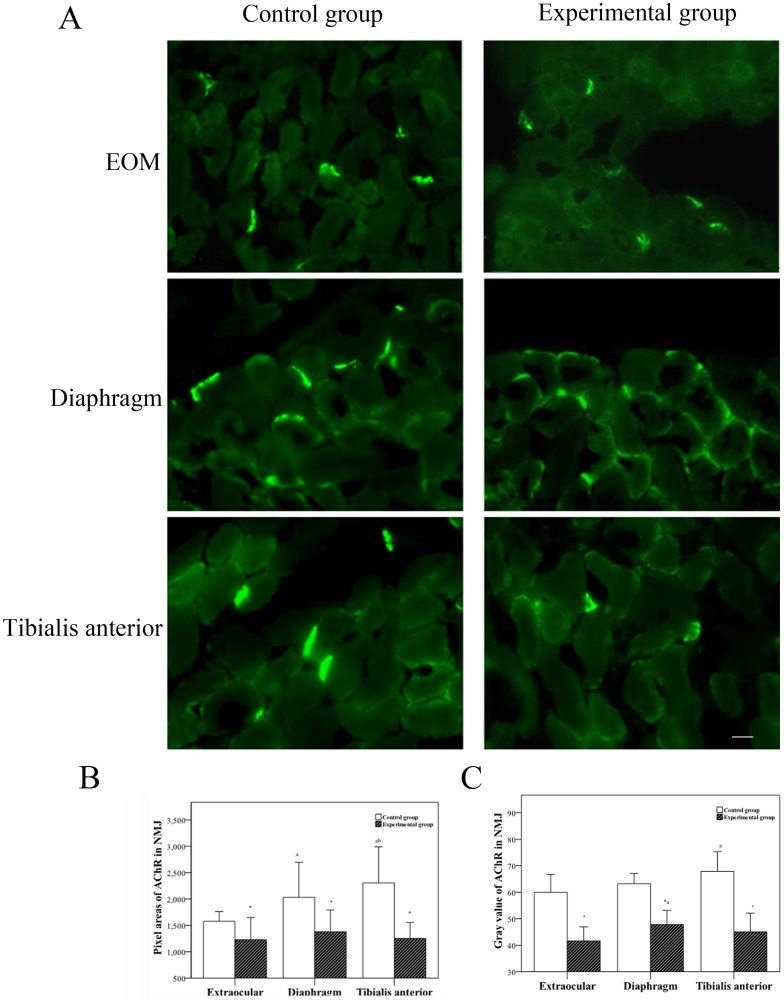
Comparision of AChRs in the NMJ of different muscles by fluorescence histochemistry. A. Acetylcholine receptors (AChRs) labeled with α-bungarotoxin in the neuromuscular junction (NMJ) of three muscles in the two groups. B. Determination of pixel areas in NMJ by an automatic analyzer. C. Determination of gray value in NMJ by an automatic analyzer. *Significant (*P*<0.05) difference between the control and experimental groups determined by independent two-sample *t* test. ^a^Statistically significant difference between indicated group and EOM group. ^b^Statistically significant difference between indicated group and diaphragm group. Scale bar = 10 µm.

Fluorescently labeled, α-BuTx–tagged AChRs aggregated in the NMJ along the border of the muscle fibers. Fluorescence intensity reflected AChR content, and fluorescence area reflected AChR distribution. In the experimental group, the fluorescence area in the different muscles was decreased when compared to control group, and this was accompanied by a decrease in fluorescence intensity (Fig. 4A). There was also a significant (*P*<0.05) difference in AChR distribution in the NMJ between the two groups. In the control group, the AChR positive area of EOM was smaller than that of diaphragm or tibialis anterior muscle (Fig. 4B). In addition, there was a significant difference between the two groups in NMJ fluorescence density of the three types of muscles (Fig. 4C).

### Comparison of the NMJ in Different Muscles by Double-Staining

**Figure 5:**
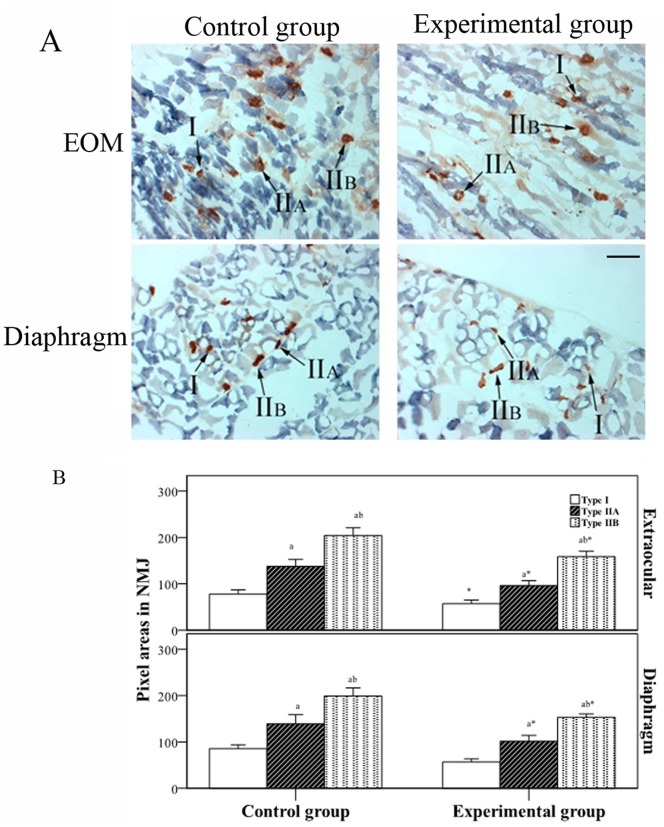
Comparision of the NMJ in different muscles by double-staining. A. SDH and AChR staining in EOM and diaphragm muscle fibers. B. Determination of pixel areas in neuromuscular junction by an automatic analyzer. *Significant (*P*<0.05) difference between control and experimental groups by independent two-sample *t* test. ^a^Statistically significant difference between indicated group and type I muscle fibers. ^b^Statistically significant difference between indicated group and type IIA muscle fibers. Scale bar = 20 µm.

Double-staining for SDH and AChE can be used to differentiate the NMJ of different muscle fibers. The background of PTMG sections in SDH-stained sections was lighter than that in control sections, which may be related to decreased SDH expression in MG. The NMJ area of the different muscles was decreased in the experimental group compared to the control group, and the NMJ area was smaller in sections containing relatively strong SDH staining. Type I muscle fibers were decreased in the experimental group compared to the control group, but the NMJ in type I muscle fibers was comparable among the different muscle types (Fig. 5A). The NMJ area in muscle fibers with relatively strong SDH staining was smaller than that in muscle fibers with weak SDH staining, which was characteristic of both EOM and diaphragm muscle (Fig. 5B).

### Comparison of AChRs in the NMJ of Different Muscles by Transmission Electron Microscopy

**Figure 6:**
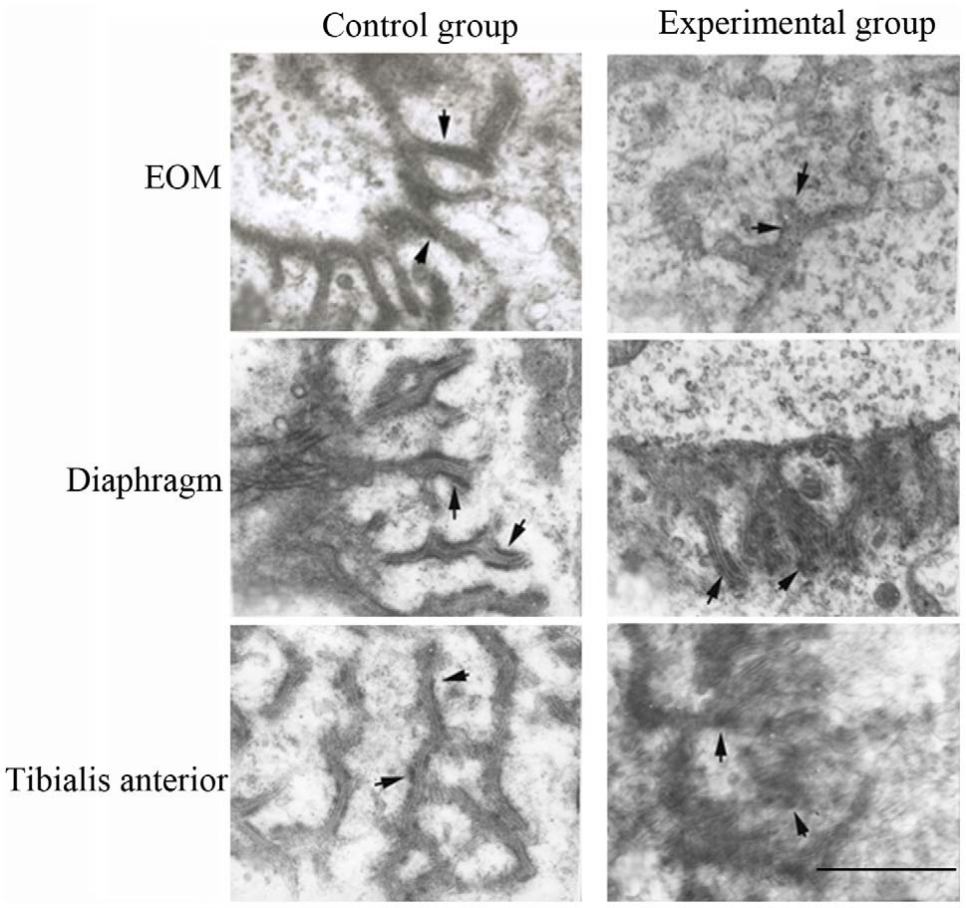
Ultrastructure of acetylcholine receptors labeled with α-bungarotoxin in the neuromuscular junction of three muscles in the two groups. Scale bar = 2 µm.

α-BuTx–labeled AChRs in the NMJ were characterized by high electron-dense material in the wrinkled surface of the postsynaptic membrane. In the experimental group, the wrinkled surface of the postsynaptic membrane was eliminated, and the AChR amount was decreased compared to that in the control group (Fig. 6). In addition, the amount of AChR in the EOM was less than that in diaphragm and tibialis anterior muscle in both groups.

### Determination of the Ratio of Postsynaptic Membrane to Presynaptic Membrane in Different Muscles

**Figure 7:**
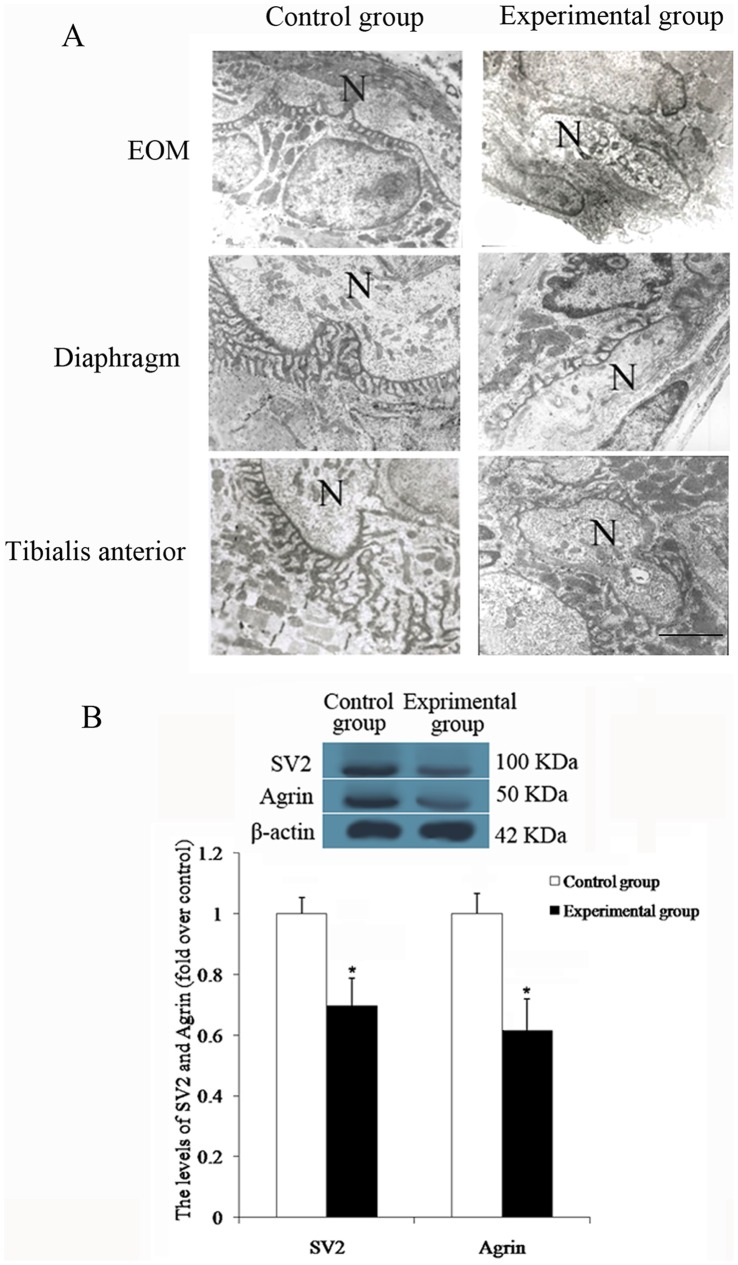
The diversity of the neuromuscular junction in three muscles in the two groups. A. Ultrastructure of the neuromuscular junction (N = nerve endings). B. Western blot analysis for Agrin and SV2 levels in EOM. *Significant (*P*<0.05) difference between the control and experimental groups determined by independent two-sample *t* test. Scale bar = 5 µm.

Normal EOM had a lower ratio of postsynaptic membrane to presynaptic membrane compared to diaphragm and tibialis anterior muscle, although the ratio of nerve terminal area to postsynaptic membrane area was greater. The experimental group exhibited a decrease in the wrinkled surface of the synapse in the NMJ of the different muscles, compared to the control group. This was accompanied by decreased number of NMJ. The ratio of postsynaptic membrane to presynaptic membrane was therefore smaller, and the ratio of nerve terminal area to postsynaptic membrane area was larger compared to the control group (Fig. 7A). Table 2 shows that the ratio of postsynaptic membrane to presynaptic membrane at the NMJ was markedly decreased in the experimental group compared to the control group, but the ratio of nerve terminal area to postsynaptic membrane area was larger than that in the control group (all *P*<0.001). In addition, the EOM of the control group had a smaller ratio of postsynaptic membrane to presynaptic membrane compared to the tibialis anterior muscle. The ratio of postsynaptic membrane to presynaptic membrane in the EOM of the experimental group was smaller than that seen in diaphragm and tibialis anterior muscle. In both groups, the ratio of nerve terminal area to postsynaptic membrane area in the EOM was larger than that in tibialis anterior muscle. Furthermore, 2 markers of pre- and postsynaptic complex of NMJ, SV2 and Agrin, were determined by Western blot. In the experimental group, the levels of SV2 and Agrin in EOM were decreased significantly when compared to the control group (Fig. 7B). The results demonstrated that presynaptic membrane and postsynaptic membrane were damaged in EOM.

**Table 2:** Comparison of post- and presynaptic membrane ratios between the two groups.

Muscle	Control group	Experimental group	*P* value
Postsynaptic membrane length/presynaptic membrane length			
Extraocular	2.76±0.98	1.55±0.37	<0.001*
Diaphragm	3.29±0.93	2.01±0.37^a^	<0.001*
Tibialis anterior	3.71±1.42^a^	2.02±0.26^a^	<0.001*
Nerve terminal area/postsynaptic membrane area			
Extraocular	0.81±0.37	1.33±0.47	<0.001*
Diaphragm	0.55±0.27^a^	1.05±0.66	<0.001*
Tibialis anterior	0.45±0.27^a^	0.90±0.25^a^	<0.001*

* Significant (*P*<0.05) difference between control and experimental groups by independent two-sample *t* test.

a Statistically significant difference between indicated group and extraocular group.

### Differentially Expressed Genes in the EOM

We showed that the experimental and control groups exhibited a total of 54 differentially expressed genes in the EOM of F344 rats. This included 25 genes that were significantly upregulated and 29 genes that were significantly downregulated. Some of these differentially expressed genes and their functions are listed in Table 3.

**Table 3:** Some of the differentially expressed genes in extraocular muscle of PTMG F344 rats.

Genebank no.	Protein name	Cy5/Cy3	Function
BI274159	Tax ion interactive protein 1	2.065	Antigen recognition, cytokine regulation
BG381611	Serine/arginine protein-specific kinase 1	2.096	Protein phosphorylation, signal transduction
AF020618	Myeloid differentiation (MyD) primary response gene 116	2.136	Cell differentiation, immune response
BE109664	Casein kinase II alpha 1 polypeptide	2.173	Protein phosphorylation, synaptic transmission
BI285643	Coenzyme Q6	2.217	Electron transport chain
NM_012497	Aldolase C	2.234	Glycolysis
BF285026	Epithelial cell-specific adhesion molecule	2.264	Cell adhesion, tissue construction
M92340	Interleukin 6 signal transduction molecule	2.315	Immune response
BI278556	Alpha-mannosidase II	2.346	Glycosylation
AA894099	Vacuolar transport protein 4a	2.559	Storage and transport of substance
NM_017266	Lactate dehydrogenase 3	2.936	Glycolysis
AI454845	Alpha-mannosidase II	3.267	Glycosylation
NM-017164	Cap protein alpha 3 (Cappa3)	0.294	Muscle contraction
AB030238	Transcription related factor in liver cancer	0.420	Cell differentiation
NM-022519	Alpha-1 protease inhibitor	0.425	Regulation of intracellular homeostasis
NM-053779	Serine protease inhibitor 1	0.458	Regulation of intracellular homeostasis
NM-013146	Calmodulin	0.460	Muscle contraction
U07201	Aspartic acid synthase	0.478	Cell metabolism
NM-031531	Serine protease inhibitor	0.495	Regulation of intracellular homeostasis
M15327	Alcohol dehydrogenase	0.499	Cell metabolism

### Differentially Expressed Genes Between EOM and Tibialis Anterior Muscle in Control F344 Rats

Microarray results showed a total of 725 differentially expressed genes between EOM and tibialis anterior muscle in control F344 rats. This included 339 genes that were significantly downregulated and 386 genes that were significantly upregulated. Some of these differentially expressed genes and their functions are listed in Table 4.

**Table 4:** Some differentially expressed genes between extraocular muscle and tibialis anterior muscle in control F344 rats.

Genebank no.	Protein name	Cy5/Cy3*	Function
AI549050	MGC72984 thymic protein 28	2.034	Immune regulation
NM_013069	Cd74 antigen	2.171	Immune response
NM_013011	Ywhaz/tryptophan oxygenase	2.205	Cell metabolism
NM_019192	Sepp1 gene	2.356	Cell differentiation
NM_012720	Mobp Myelin-binding protein	2.500	Development and differentiation
NM_024488	Cdk5rap3, Cdk5 regulatory protein	2.511	Signal transduction
AF139830	Igfbp5 insulin-like growth factor binding protein	2.538	Homeostasis regulation
AF065438	Lgals3bp lectin sugar-binding protein	2.655	Immune regulation
BE107386	Death polypeptide 27	3.013	Apoptosis
M15481	Igf1 insulin-like growth factor 1	3.072	Cell metabolism
NM_021740	Ptma thymosin	3.157	Immune regulation
NM_031509	Gsta5 Glutathione S-transferase 1	3.190	Cellular oxidative damage
BI284980	CD9 antigen	3.208	Antigen recognition, immune response
D88250	C1s complement 1	3.499	Immune response
NM_013053	YwhaqTyrosine/tryptophan oxygenase actin	3.692	Cell metabolism
NM_022592	Tkt transketolase	4.074	Cell metabolism
AI176565	Acetyl-CoA synthase 2	4.075	Cell metabolism
NM_033539	Eef1a2 transcription elongation factor	4.088	Hereditary and metabolism
NM_031811	Taldo1 transaldolase 1	4.270	Cell metabolism
AW921736	Acetyl synthase 3	4.629	Cell metabolism
NM_053445	Fads1 lipolytic enzymes	4.705	Homeostasis regulation
AI236953	Sptlc1 serine transferase long-chain	4.746	Cell metabolism
NM_012998	P4hb Hydroxylase	4.834	Cell metabolism
BG380277	Complement B-factor, properdin	4.913	Antigen recognition, immune response
AW916669	B2m microglobulin	5.295	Homeostasis regulation
NM_012595	Ldhb lactate dehydrogenase B	5.667	Cell metabolism
AA945149	Glutathione transferase	5.816	Cell metabolism
BE096678	ATP coalition 3	5.904	Oxidative phosphorylation
AI013913	Cald1	6.060	Muscle contraction
BI282044	Slc33a1 acetylcholine transporter	6.193	Muscle contraction
M25732	Pam glycosylation monooxygenase	6.298	Cell metabolism
NM_031797	Kai1: Kangai 1	6.781	Immune response
AI232065	Rho GTPase activator protein	6.785	Signal transduction
AW921149	C2 complement	7.210	Immune response
BE111695	T cell activation GTPase	7.225	Homeostasis regulation
U75903	Ugt1a6: UDP glucose transferase	7.360	Cell metabolism
AI411279	Aldehyde oxidase A1	8.560	Cell metabolism
NM_031325	UDP glucose dehydrogenase	10.025	Cell metabolism
NM_053607	Acsl5Acetyl synthase long chain	11.296	Cell metabolism
NM_022519	Serpina1 serine protease inhibitor	11.437	Cell metabolism
J03752	Glutathione S-transferase 1	12.569	Cellular oxidative damage
M15327	Adh1 Alcohol dehydrogenase	14.055	Cell metabolism
AI412189	Igha immunoglobulin heavy chain peptide	14.945	Immune response
L22655	Acetylcholine receptor antibody kappa chain	28.375	Immune response
AA817761	Adh1 Alcohol dehydrogenase 1	37.020	Cell metabolism
AF208125	Grlacs acetyl coenzyme A synthetase	76.805	Cell metabolism
NM_013146	Cald1	76.925	Muscle contraction
BG378588	Myosin binding protein C	0.078	Muscle contraction
NM_012495	Aldoa aldolase A	0.079	Cell metabolism
BI277042	Gpd1 Glycerol triphosphate dehydrogenase 1	0.085	Cell metabolism
NM_053297	Pkm2 Pyruvate kinase	0.090	Cell metabolism
BI289527	Tropomyosin 4	0.118	Muscle contraction
NM_017025	Ldha lactate dehydrogenase A	0.123	Cell metabolism
NM_058213	Rabep2, GTPase binding protein	0.130	Signal transduction
BI276959	Popdc2 cytochrome oxidase assembly protein	0.131	Oxidative phosphorylation
NM_017008	Gapd glycerol triphosphate aldehyde dehydrogenase,	0.133	Cell metabolism
BE106404	Phosphoglycerate kinase 2	0.135	Cell metabolism
NM_019212	Eno3 enolase III	0.142	Cell metabolism
M34136	Tpm1 tropomyosin	0.146	Muscle contraction
J02811	Ampd1 adenosine dehydrogenase	0.152	Cell metabolism
NM_022499	Pvalb small albumin	0.181	Homeostasis regulation
BI279760	Pgk1 phosphoglycerate kinase 1	0.181	Cell metabolism
BF284311	Immunoglobulin domain	0.182	Immune response
NM_012605	Myl2 myosin light chain 2	0.184	Muscle contraction
BG672056	Bpgm phosphoglycerate mutase	0.184	Cell metabolism
U22893	Csda cold shock protein	0.188	Homeostasis regulation
BG671533	ADP transferase 3	0.192	Oxidative phosphorylation
AI412065	Aldehyde reductase	0.210	Cell metabolism
NM_054006	Unr: Unr protein	0.219	Stress
AI171098	Mybpc1 myosin binding protein	0.222	Muscle contraction
NM_053638	Idh3a isocitrate dehydrogenase3	0.235	Cell metabolism
NM_012606	Myl3 myosin	0.253	Muscle contraction
NM_019132	Gnas: GNAS gene	0.292	Endocrine regulation
AA851682	Sell seletin	0.294	Immune response
BG666669	UDP glucose pyrophosphorylase	0.313	Cell metabolism
BE113599	Abcc9,ATP binding protein	0.332	Oxidative phosphorylation
X15030	Cox5a cytochrome oxidase	0.334	Cell metabolism
BG666732	COP9 gene	0.346	Proteolysis, cell metabolism
NM_031043	Glycogen protein 1	0.351	Cell metabolism
AW919081	G6pc3: Glucose-6-phosphatase	0.355	Cell metabolism
U78977	Atp9a: ATPase	0.359	Oxidative phosphorylation
NM_031345	Dsipi sleep-inducing factor	0.369	Immune response
NM_012594	Lalba lactalbumin	0.374	Immune regulation
NM_013172	Myf6 myosin	0.383	Muscle contraction
BI296716	Acetyltransferase 5	0.393	Cell metabolism
BI274030	Syn2 synaptophysin	0.398	Signal transduction
NM_031533	Ugt2b glycosyltransferase	0.406	Cell metabolism
NM_053848	Opcml adhesion molecule	0.449	Homeostasis regulation
NM_053420	Bnip3 adenovirus-binding protein	0.466	Immune regulation
BF285164	COX17 cytochrome oxidase	0.480	Oxidative phosphorylation
AI412014	Mitochondrial transporter 2	0.488	Cell metabolism
BG671518	Serine threonine kinase receptor-related protein	0.494	Cell metabolism

* Cy5/Cy3 refers to the ratio of extraocular muscle to tibialis anterior muscle.

## Discussion

In this study, we investigated EOM susceptibility in MG. We used a PTMG rat model based on previous reports that in PTMG, electrophysiological and ultrastructural changes at the endplate are similar to those seen in patients with acquired MG [14]. We showed that the NMJ in the EOM of PTMG rats was simpler in structure compared to NMJ structures in the diaphragm and tibialis anterior muscle. This was manifested by fewer synaptic folds in the postsynaptic membrane, and fewer AChRs. Although passive transfer studies have been previously used in mice, we used a rat model because the disease features are more obvious in rats and because mAb35 is a rat antibody. In addition of using mAb35, we plan to use an irrelevant mAb in future studies to serve as an additional control.

We used SDH staining to show a significant decrease in type I muscle fibers and a significant increase in type IIA muscle fibers in PTMG rats compared to control rats. The extent of change differed among the different muscles. The proportion of type IIB muscle fibers remained largely unchanged. Our data suggested that the skeletal muscle weakness, resulting from NMJ transduction disorder, could be due to a decrease in fatigue-resistant, oxidative type I fibers. Decreased SDH expression in the muscle cells could reflect a transformation of type I fibers with high SDH expression into type IIA fibers with low SDH expression. Interestingly, the diaphragm and tibialis anterior muscle had a smaller proportion of type I fibers compared to the EOM. This may be related to the higher proportion of type I fibers in normal EOM or to the different loads in distinct muscles.

We showed a decrease in the AChE-positive area in all three muscles in the experimental group compared to the control group, although the AChE density remained unchanged. Since AChE is mainly located in the basement membrane of the synaptic space of the NMJ, PTMG rats exhibited a decrease in the area of the synaptic space (area of the basement membrane), although AChE content in the synaptic space remained unchanged.

We used AChE staining to show that NMJ size was similar in the EOM and in tibialis anterior muscle. We also used α-BuTx–labeled AChRs to show a significant decrease in the amount of AChR in the NMJ of PTMG rats compared to control rats. EOM had a lower AChR density compared to the other two muscles in both groups, although this was more obvious in the PTMG group. The EOM of the control group also had significantly decreased AChR–positive area compared to the other two muscles, although no marked difference was found among the different muscles in the experimental group. The small amount of AChR in the NMJ makes the EOM susceptible to immune attack in the presence of even low titers of antibody.

It was previously suggested that the length of the synaptic fold in unit area could reflect the amount of voltage-dependent sodium channels and AChRs [15]. Consistent with earlier reports [16], we showed that the NMJ structure in the majority of the EOM was simpler than in the other two muscles. In the experimental group, only a few synaptic folds were found in the postsynaptic membrane, which was almost flat compared to normal EOMs where a complex synaptic structure was observed. Although the EOM and other skeletal muscles were involved in MG, the susceptibility of the EOM differed from that of the other muscles. Following attack by the immune system, the tolerance of the EOM becomes relatively poor, although changes in the types of different fibers, AChR amount, and NMJ ultrastructure were similar among the three muscles.

We showed that the EOM endplate was wider than that of the diaphragm or tibialis anterior muscle. The length of the muscle fiber in the EOM was one-tenth that in the other skeletal muscles, and muscle fiber diameter in the EOM was half that in the other skeletal muscles. The larger ratio of endplate to muscle fiber reflects the fact that movement of the EOM is finer than that of the other muscles.

Although the ratio of postsynaptic membrane to presynaptic membrane in the EOM was significantly decreased compared to the other muscles in both groups, EOM had a significantly higher ratio of nerve terminal area to postsynaptic membrane area compared to the other muscles. Since the length and area of the postsynaptic membrane reflect the amount of voltage-dependent sodium channels and AChRs, our data suggested that the NMJ of the EOM had fewer sodium channels and less AChRs than the other muscles. Release of synaptic vesicles from the presynaptic membrane would result in decreased amount of sodium channels and decreased action potential. The decreased difference between action potential and membrane potential would lead to a low safety factor.

Expression profiling previously showed 338 genes that were differentially expressed in the EOM compared to quadriceps femoris limb muscle [17]. In the present study, we showed that immune response genes such as thymosin, CD9, CD74, C1, C2, and B factor were significantly upregulated in the EOM compared to that in tibialis anterior muscle. The immunoglobulin domain gene, AChR antibody kappa chain gene was also upregulated. We also showed downregulation of genes associated with neuromuscular synaptic transmission including myosin, tropomyosin, myosin-binding protein, and synaptophysin and upregulation of ACh metabolism-related acetyl synthase and ACh transporter genes. Genes for ACh metabolism-related acetyltransferase, acetyl reductase, and mitochondrial transporter were also downregulated. In addition, some cell metabolism and signal transduction–related genes such as Rho, CDK5, and genes encoding proteins in the respiratory chain were differentially expressed. We would like to elucidate the role of these genes in the susceptibility of the EOM to MG. Of note, genes for complement regulatory factors such as CD55 (decay-accelerating factor; DAF) and CD59 remained unchanged between control rats and PTMG rats, although both have been previously found to be closely related to the susceptibility of the EOM to MG [18]–[23]. A single nucleotide polymorphism in the DAF regulatory region was previously shown to be associated with EOM paresis [23]. It will be interesting to evaluate gene polymorphisms in some of the differentially expressed genes in our study and explore their role in EOM paresis. Our study provides the basis for further investigation of the mechanisms underlying increased EOM susceptibility in MG.
